# Affective Spectrum Disorders in an Urban Swedish Adult Psychiatric Unit: A Descriptive Study

**DOI:** 10.1155/2012/527827

**Published:** 2012-03-28

**Authors:** M. Scharin, T. Archer, P. Hellström

**Affiliations:** ^1^Department of Psychology, University of Gothenburg, 417 57 Gotheburg, Sweden; ^2^Unit for Neuropsychology and Neuropsychiatry, Sahlgrenska University Hospital, Gothenburg, Sweden

## Abstract

*Background*. Several studies have found that patients with affective-/anxiety-/stress-related syndromes present overlapping features such as cooccurrence within families and individuals and response to the same type of pharmacological treatment, suggesting that these syndromes share pathogenetic mechanisms. The term affective spectrum disorder (AfSD) has been suggested, emphasizing these commonalities. The expectancy rate, sociodemographic characteristics, and global level of functioning in AfSD has hitherto not been studied neglected. *Material and Method*. Out of 180 consecutive patients 94 were included after clinical investigations and ICD-10 diagnostics. Further investigations included well-known self-evaluation instruments assessing psychiatric symptoms, personality disorders, psychosocial stress, adaptation, quality of life, and global level of functioning. A neuropsychological screening was also included. *Results*. The patients were young, had many young children, were well educated, and had about expected (normal distribution of) intelligence. Sixty-one percent were identified as belonging to the group of AfSD. *Conclusion*. The study identifies a large group of patients that presents much suffering and failure of functioning. This group is shared between the levels of medical care, between primary care and psychiatry. The term AfSD facilitates identification of patient groups that share common traits and identifies individuals clinically, besides the referred patients, in need of psychiatric interventions.

## 1. Introduction

The organization of psychiatric treatment, following diagnosis, follows an ordering of treatment (1°-2° care) within specialization, child psychiatry, forensic psychiatry, and general psychiatry, subspecialization, psychoses, and affective disorders, all of which facilitates general observations of the patient population. Nevertheless, a consideration of those presenting depression, anxiety disorders, personality disorders (most commonly Clusters B and C, DSM-IV [1]), and stress-related conditions shows minimal analysis beyond diagnosis and treatment cost, thereby precluding realistic goals, treatment designs, and evaluation. Although investigations of pure diagnostic groups reduce confounding variables, constraints on ecological validity, this is the clinical setting of specialized outpatient units (SOUs), imply requirements for neuroscientific assessment of patients under these conditions in combination with epidemiological data unfettered by admittance selection based on symptom severity and complexity, incidence, and prevalence (etiopathogenesis and socioeconomics).

It has been suggested that 14 psychiatric and medical disorders may share a specific (as yet unknown) neurophysiologic, etiologically specific abnormality: Affective Spectrum Disorder (AfSD), including 10 psychiatric conditions (attention-deficit/hyperactivity disorder, bulimia nervosa, dysthymic disorder, generalized anxiety disorder, major depressive disorder, obsessive-compulsive disorder, panic disorder, posttraumatic stress disorder, premenstrual dysphoric disorder, and social phobia) and four medical conditions (fibromyalgia, irritable bowel syndrome, migraine, and cataplexy) [2–6]. The spectrum may involve four or more subgroups: (i) low or dysphoric mood, including diagnoses of major depression and dysthymic, even bipolar disorder I and II, (ii) anxiety-ridden patients, presented by general anxiety, social phobia, panic disorder, OCD, even other anxiety disorders, as well as anancastic and avoidant personality traits, (iii) impulse control difficulties, ADHD, eating and gambling disorders, and borderline traits, and finally (iv) a heterogeneous subgroup defined by vulnerability to chronic/severe stress including PTSD, “burnout,” or “fatigue syndrome” (coded in ICD 10 [7] as F43.8A: “other reactions to severe stress”) and constituting psychosocial stress and psychiatric symptom associations with neuropsychological symptoms of lack of concentration and memory problems. Another subgroup consists of patients presenting signs of somatization or bodily complaints like fibromyalgia, irritable bowel syndrome, cataplexy, and migraine, generally categorized as comorbidities.

In addition to parsimony and clinical and basic utility, AfSD captures multiple expressions, from eating disorders to major depression, and accounts for several confounding traits and great cooccurrence in the group. The constituent diagnoses may represent different phenotypes but a common genotype. The concept and the constituents of AfSD are presented in Table 1.

The related notion, Affective Spectrum Psychosis (ASP), distinguishes bipolar disorder with psychotic features and major depressive disorder with psychotic features, implying that patients with affective psychosis share many traits and characteristics with patients with schizophrenia spectrum disorder [9]. Here, manic delusion presents a mood congruent exaggeration rather than schizophrenic type of delusion. The present account, first, by the reintroduction of the readily applicable notion of AfSD, a novel research focus with symptoms rather than diagnosis as starting point, is maintained. Second, a comprehensive description of patients consecutively admitted to our SOU during the first year of NU (new admissions and followup) is offered. Third, the present account demonstrates how responsibility for this group of patients is divided between primary care and psychiatry in Sweden with the implication that the majority of these patients will have long periods of sickness benefits within the social service.

## 2. Patients and Methods

**Figure 1:**
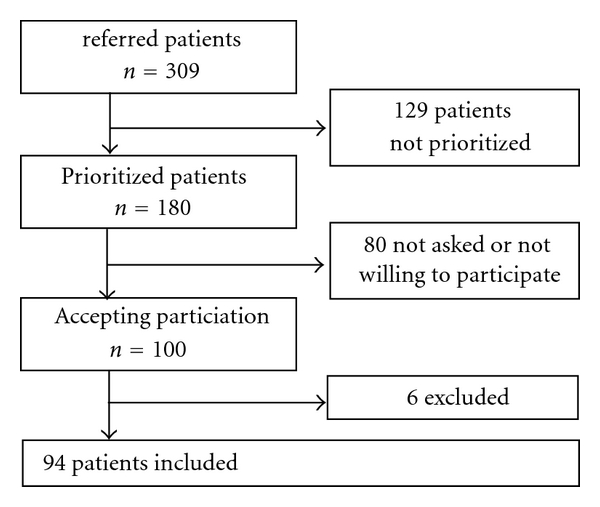
Flow chart depicting extraction of the 94 eligible patients observed in the study.

All patients referred to Lergöksgatan SOU during 2001 were considered for inclusion. The catchment area has 50 000 inhabitants and three primary care units. The unit is part of general psychiatry and receives all admissions except for patients with primarily psychosis (schizophrenia and paranoid psychosis). During this period, the SOU received 309 referrals, the majority of which came from the primary care units. Of these, 180 cases were allowed admittance to the SOU for clinical investigation and treatment consideration and thus preliminarily eligible for this study. During a same period of time 6 patients with suspected psychotic symptoms were investigated by the neighboring SOU, specialized on these conditions. One hundred patients accepted to participate. Six patients were excluded due to diagnoses of schizophreniform or schizoaffective syndromes or substance abuse, that is, disorders belonging to other units according to the subspecialization in the region (see Figure 1). Ninety-four patients, were subsequently diagnosed with depression (*n* = 29), anxiety (*n* = 28), neuropsychiatric disorders (*n* = 9), stress syndromes (*n* = 6), burnout (*n* = 6), and bipolar disorder (*n* = 6). Personality disorders (*n* = 5), and somatoform syndrome (*n* = 3), eating disorder (*n* = 1), and organic syndrome (*n* = 1) were included in the study and further investigated as shown below.

The information collection was carried out by the professional assigned to the patient for the first encounter clinical interview: (psychiatrist (50%), clinical psychologist, psychiatric nurse, or counseling therapist). All instruments, but two, are Swedish, and validated on Swedish population samples. SF 36 has had an ambitious validating procedure [10], whereas SASS is not validated in Sweden.

### 2.1. Sociodemographic Characteristics

At the initial visit patients were asked to give a description of basic living arrangements.

### 2.2. Psychiatric Diagnosis including Diagnostic Instruments

The patients were diagnosed according to ICD 10 criteria [7] by specialized psychiatrists. The diagnosis was based on information received from the interview with the patients, and the following diagnostic tools: the Comprehensive Psychopathological Rating Scale, Self-rating Scale for Affective Syndromes (CPRS-S-A, [11]) consisting of 19 self-rated variables that correspond to three scales that are commonly used for rating of depression (Montgomery Åsberg Depression Rating Scale, MADRS [12]), anxiety (Brief Scale for Anxiety, BSA [13]), and obsessive-compulsive symptomatology (CPRS-OCD-scale [14]). In the Swedish version of MADRS values between 0 and 12 are considered normal, whereas higher scores reflect increasingly severe depressive symptoms. The anxiety scale is also an ordinal scale, but lacks corresponding limits for grading of severity. In the OCD-scale, the patient admits to or denies the presence of core symptoms of obsession or compulsion, making the scale nominal rather than ordinal.

### 2.3. General Level of Intellectual Functioning

The cognitive level was assessed with a short version of the WAIS-R (Wechsler Adult Intelligence Scale-Revised [15]), including two verbal (Information and Digit Span) and two performance tests (Block Design and Digit Symbol). The results on these four tests were used as an approximation of the full scale results, a method that has been described and validated elsewhere [16].

Only the WAIS-R results of patients originating from Europe are presented, since performance of patients with other ethnic background would require specific analyses.

### 2.4. Personality

The DSM-IV and ICD-10 Personality Questionnaire (DIP-Q, [17]) was used to assess personality disorders. The DIP-Q contains 140 items in total, 135 of which are closely linked to diagnostic criteria. A five-item impairment and distress scale (ID-scale) and a self-report version of the scale for Global Assessment of Functioning GAF [18] are also included in the questionnaire.

In psychiatry (medicine) personality has a different status than in psychology. In psychiatry we screen for traits that are known to create “adjustment problem.” Eleven traits are described in DSM-IV [1]. The Axis II personality disorders are generally grouped into three clusters. Even though there is no evidence that the personality statistically congregates into these clusters, this notion seems reasonable intuitively on the basis of clinical realities.

The clusters get alphabetical names: cluster A describes odd and eccentric disorders and includes schizoid, schizotypal and paranoid personality disorders. Cluster B covers dramatic, emotional, or erratic disorders and includes borderline personality-, histrionic-, and antisocial personality disorders. The anxious or fearful disorders were previously named neurotic disorders and represent Cluster C (avoidant, anancastic (Obsessive-compulsive)). (Some preclude that also a dysthymic personality trait might be added, but this is yet not agreed.)

The professionals were instructed to avoid screening for personality disorders when the patients were acutely ill.

### 2.5. Global Level of Functioning and Psychosocial Stress

The Global Assessment of Functioning Scale (GAF, [19]) is a measure of psychological, social, and working level of functioning in a hypothetic continuum of mental health—disability. The interval between 41 and 50 is referred to as severe, 51–60 as moderate, and 61–71 as mild symptoms and reduction of level of functioning.

GAF (the axis V in the DSM system of diagnosis) has become the major instrument in Swedish psychiatry, to discuss levels of healthcare. Both symptom pressure and functional appreciation are fused together to a single point of measure, rendering the measure a controversial issue in psychiatry.

**Psychosocial Stress**

The scale of psychosocial stress is included in DIP-Q. Stress is scored as present or absent in eleven life domains and can be scored in a yes or no fashion. The experienced stress is scored on a six-graded scale from no, to catastrophically.

### 2.6. Social Functioning

Social Adaptation Self-evaluation Scale (SASS) [20] was used to asses social functioning and role fulfillment at work, in social activities and in close relationships [21]. Values between 35 and 52 are considered normal [20]. SASS is not yet validated in Sweden.

### 2.7. Health-Related Quality of Life

The Short Form 36 Questions health status instrument (SF36) [10] was used to assess health-related quality of life.

The study has been approved by the Ethics Committee of Gothenburg with the reference number S 225-00.

## 3. Results

### 3.1. Sociodemographics

The sociodemographic data are presented in Table 2.

### 3.2. Medication

At the time of the initial contact most patients had had previous contact with health professionals and a many of them (75%) were on medication (Table 3).

### 3.3. Psychiatric Symptoms and Diagnoses

We can conclude that 61% of the group can be organized under the heading of affective spectrum disorder strictly according to Hudson and Popes definition and 87% according to this paper's speculation. Somatoform syndrome, that is, chronic pain, can be placed under the heading of fibromyalgia. Schizoid symptoms, schizoaffective syndrome, and neuropsychiatric disorders other than ADHD and organic syndromes fall outside the AfSD grouping.

Half of the patients respond to CPRS-S-A form in a manner that corresponds to depression. Panic disorder (14%) and/or symptoms of compulsion (12%) were also frequent.

### 3.4. Personality Diagnoses

Sixty-four percent fulfilled criteria for any personality disorder according to DIP-Q, ICD 10. A large proportion met criteria for multiple domain personality disorders. As many as 27% met criteria in all three clusters of personality disorders according to the personality disorder clusters of DSM-IV. Odd and eccentric disorders together with anxious or fearful disorders (Clusters A and C) were seen in 19%. Patients within Cluster C were 12% and the combination between dramatic, emotional, or erratic disorders (Cluster B) and Cluster C were found in 3%. It was unusual to meet criteria in Cluster A alone (2%).

### 3.5. Health-Related Quality of Life

The quality of life is severely affected in the group. This is especially evident on the scales measuring vitality, emotional role, and physical role (see Table 4).

### 3.6. GAF (Global Assessment of Functioning)

On the overall rating of global level of functioning, 62 percent of the surveyed patients estimated level of GAF above the threshold 50.

### 3.7. Psychosocial Stress

Just one-tenth of the patients reported no actual life stressor. The group reported multiple problematic life circumstances; the mean number of the group was three life stressors. As many as one-fourth of the patients reported loss of a loved one (23%). The most reported problem was difficulties at work (65%) or studies (38%) (some patients were involved in both studies and work). Sixty-one percent reported family problems and 38% reported economic problems and/or housing problems. Twenty-five percent reported problems with the medical care worth considering. War, disaster, or serious accidents were reported by 11% of the cases. The same proportion of patients described problems with the juridical system (11%). The severity of the stress generated by these events is presented in Table 5.

### 3.8. Intellectual Functioning

At large, the survey shows that the group studied has an expected distribution, that is, similar to that of healthy individuals in normative studies. However, a slightly larger proportion of patients with exceptionally low level of functioning (IQ < 70) was found and is 8 instead of the expected approximately 2%. Forty-nine percent of the patients were in the average (90–109) and 23% in the higher and lower ranges.

In the drop-out analysis we can observe that 48% of the nonincluded group (from the 180) came just for consulting (1–4 visits) compared to 16% in the included group. The rest of the non-included group does not differ in any of the investigated variables (gender, age, symptom profile, or diagnosis).

## 4. Discussion

The main findings of this study are that in a group of consecutive patients referred to an outpatient clinic a majority (61%) presented symptoms corresponding to AfSD or 87% according to the extended concept. It ought to be noted that the methods applied enhanced the investigative and diagnostic work and the treatment evaluation. GAF (Global Assessment of Functioning, axis V in DSM-IV) is the main instrument referred to in the communication between the SOUs and primary care, differentiating between primary care and psychiatry. GAF scores at or below 50 require psychiatric investigation and treatment [22], except in cases with psychoses or a bipolar disorders which are always the responsibility of specialist psychiatry, regardless of GAF score. Similarly, investigations of suspected neuropsychiatric disorders are always to be performed by the SOUs.

### 4.1. Division between Primary and Specialist Care

The present study shows that primary care and psychiatry share the same patients at different times during the course of the illness. The AfSD concept will assist primary care in its commitment to the patient group. With extreme illness requiring 24-hour care, responsibility rests with psychiatry. When the patient has recovered to a more normal level of functioning, psychiatry refers the patient to primary care. In the intermediary period, the SOUs have the responsibility of restoring the patients' level of functioning.

### 4.2. The Concept of AfSD Is Useful Both in Primary Care and Psychiatry

The most extensively studied diagnosis under the umbrella of AfSD is without doubt major depression. Clinically, it seems that all forms of AfSD might benefit from treatment not just in a palliative sense, but by fundamentally interrupting the chain of etiologic steps associated with the disorder [3], for example, treatment of chronic pain with antidepressants might halt the disorder at its core. If investigation and treatment of AfSD were to be dealt with as vigorously as major depression is today, that would prove beneficial for clinical practice. In treatment protocols for depression, different recommendations are provided, depending on the severity of the condition (minor, moderate, and severe) and on specific features of the syndrome (somatic signs, bipolarity, etc.). Recognition of different AfSDs and provision of specific guidance for specific disorders implement both theoretical understanding and clinical treatment; although pharmacotherapy may be similar, psychotherapeutic interventions may vary. Due to either side effects or lack of effectiveness, initial pharmacological treatment of depression produces unsatisfactory results in approximately one-third of the patients. In this group, there is a need to change to or complement with psychological treatment [23].

### 4.3. Detection of AfSD

Only about half of depression sufferers are generally detected in primary care, generally the most severe cases. It is safe to say that AfSD at large is more difficult to detect than the more well-known diagnosis of depression. Simple questionnaires such as the ones used in this study that are either completed by patients or used by doctors during an appointment may help identify a larger proportion of these patients. Multicentre primary care studies show that when patient instruction, telephone support and computerized reminders about treatment protocols are offered, as well as ready access to psychiatrists and psychologists trained in short-term psychotherapy, and this is preferable to routine medical care [23]. 

### 4.4. Genetic Vulnerability

There is strong evidence that burnout, “fatigue depression,” and major depression can be associated with genetic factors. In a study by Kilpatrick et al. [24] the low-expression variant of the 5-HTTLPR polymorphism (the short version) was shown to increase the risk of so called “post hurricane” posttraumatic stress disorder (PTSD) and major depression under conditions of high hurricane exposure and low social support, after statistical adjustment for sex, ancestry, and age. Similar effects were found for major depression. High-risk individuals were at 4.5 times the risk of developing PTSD and major depression compared to low-risk individuals.

### 4.5. Impact of AfSD

The quality of life is severely affected in the studied group of patients. That vitality (subjective feeling of energy and power) and emotional role (the capacity to perform important activities as parent or wife/husband) are severely affected among patients suffering from AfSD symptomatology comes as no surprise. That the patients report low functioning also in the physical role (in physical/technical tasks) without reporting physical symptoms was less expected. A preliminary interpretation might be that this is an expression of the conative symptoms (serious halting of motivation and willpower/desire) often seen in depression.

### 4.6. Limitations of the Study

The proposed AfSD model contains four groups of diagnoses characterized by dysphoria, anxiety, impulsivity, and experienced stress and bears the promise of enhancing our understanding of these conditions. However, it is, as yet, tentative and requires further exploration and validation.

Secondly, the use of self-administered instruments may be questioned. This study relied heavily on such instruments with two exceptions: the psychiatric diagnosis and the neuropsychological screening. There is some evidence that the patients, in severe states, tend to slightly overestimate the affliction in comparison to expert raters [25]. Yet, which expression is the more accurate? We have chosen to rely on the patients rather than the experts because the patients remain constant whereas the experts differ widely in experience and training, rendering comparisons between expert ratings uncertain. If the patient does the work comparisons may prove more accurate [26].

## 5. Conclusion

This study identifies a large group of patients that has undergone great suffering, a high level of psychosocial stress, and a low level of functioning and quality of life. This group is shared between the primary care and psychiatry.

The term AfSD facilitates identification of these patients who, despite different diagnostic labels, do share important common traits; for example, they do respond to the same pharmacological treatment, the disorders commonly occur in the same individual (cooccurrence), and their children and relatives tend to show related symptoms (co-aggregation). The concept has provided clinical notions and an identification of individuals in need of treatment in a familial context.

## Figures and Tables

**Table 1:** Schematic model of Affective spectrum disorders (AfSDs), an interpretation and expansion of the concept as discussed in [2–6, 8].

Dysphoria	Anxiety	Impulse	Stress
Major depression*	Generalized anxiety*	ADHD*	PTSD*
Dysthymic disorder*	Social phobia*	Bulimia nervosa*	Burnout syndromes (fatigue syndrome)***
Bipolar disorder I**	Panic disorder*	“Binge-and-purge” eating disorder*	
Bipolar disorder II**	OCD*	Borderline personality disorder***	
	Anancastic and avoidant personality disorder***		
Bodily complaints			
Fibromyalgia, irritable bowel syndrome, cataplexy, migraine*			

*Hudson and Pope [2–6], **[8], ***authors' inference.

**Table 2:** Sociodemographic description of the included patients (*n* = 94). All numbers are percentages.

Gender		Children	
Male/female	37/63	Yes/no	65/35
Age		Education	

18–29	28	Unfinished primary school	5
30–39	31	Primary school (=9 years)	19
40–49	18	Secondary or vocational school	47
50–59	11	University	26
≥60	12	No information	3

Accommodations		Income	

Apartment	51	Salary/student loan	38
Villa/house	26	Sickness benefit	32
Tenant	10	Pension	12
No information	13	Social/unemployment benefit	9
		Other	5
		No information	4

Marital status		Decent	

Married/stable living arrangements	45	Born in Sweden, no foreign decent	73
Single	28	Born in Sweden, foreign decent	8
Divorced/living apart from partner	20	Foreign-born, Europe	9
Widow/widower	3	Foreign-born, the orient	9
No information	4	Foreign-born, other	1

**Table 3:** Previous treatment and ongoing medication (*n* = 94). Numbers are percentages.

Previous treatment		Current medication	
None specified previous contact	23	Any medication	75
Physician	52	SSRIs	42
Psychologist	33	Tranquilizer	13
Physiotherapist	15	Sleep medicine	12
Counselor	17	Pain medicine	9
Nurse	5	Antipsychotic medication	5
Occupational Therapist	6		
Other	8		

SSRI: selective serotonin reuptake inhibitor.

**Table 4:** Quality of life according to SF 36 (*n* = 91).

Quality of life SF 36 *n* = 91	Md (range)
Physical function (PF)	85 (0–100)
Role physical (RP)	25 (0–100)
Bodily pain (BP)	41 (0–100)
General health (GH)	42 (5–97)
Vitality (VT)	25 (0–80)
Social function (SF)	38 (0–100)
Role emotional (RE)	0 (0–100)
Mental health (MH)	32 (0–92)

**Table 5:** The severity of stress generated by stressful life events (axis 4, DSM-IV).

Severity of stress	
*n* = 85	**%**
Severe	33
Moderate	27
Extreme	22
None/light	8
Catastrophic	6
